# Effects of *Yinchenhao Tang *and related decoctions on DMN-induced cirrhosis/fibrosis in rats

**DOI:** 10.1186/1749-8546-3-1

**Published:** 2008-01-31

**Authors:** Cheng Liu, Mingyu Sun, Lei Wang, Gaoqiang Wang, Gaofeng Chen, Chenghai Liu, Ping Liu

**Affiliations:** 1 Institute of Liver Diseases, Shuguang Hospital, Shanghai University of Traditional Chinese Medicine, Shanghai 201203, China; 2 Shanghai University of Traditional Chinese Medicine, Shanghai 201203, China; 3 E-institutes of Shanghai Municipal Education Commission, Shanghai University of Traditional Chinese Medicine, Shanghai 201203, China

## Abstract

**Background:**

Chinese medicine decoctions such as *Yinchenhao Tang *(*YCHT*), *Xiayuxue Tang *(*XYXT*), *Huangqi Tang *(*HQT*), *Yiguan Jian *(*YGJ*) and *Xiaochaihu Tang *(*XCHT*)) were used to treat liver cirrhosis. The present study evaluates the effects of these decoctions on fibrosis in rats induced by dimethylnitrosamine (DMN).

**Methods:**

DMN solution (0.5%) was injected to rats for three consecutive days per week for four weeks. At the beginning of week 3, rats were randomly divided into 4-week DMN control group, *YCHT*, *XYXT*, *HQT*, *YGJ*, *XCHT *and vehicle groups. Each group was orally administered with specific decoctions daily for two weeks. Rats in the vehicle group were orally administered with only water.

**Results:**

Liver fibrosis and cirrhosis were observed in weeks 2 and 4 in DMN-intoxicated rats. Compared with normal rats, alanine transaminase (ALT), aspartate transaminase (AST), alkaline phosphatase (ALP) activities and level of total bilirubin acid (TBA) in serum and content of Hydroxyproline (Hyp) in liver tissue of model group rats rose significantly. However, the albumin (Alb) level in serum decreased significantly. Compared with the 4-week DMN group, the pathological conditions and functions of the liver in the *YCHT *group improved significantly, and the content of Hyp decreased remarkably: only one rat in this group developed liver cirrhosis and the ratio of cirrhosis was only 8.3%. On the other hand, the other decoctions did not show remarkable effects. *YCHT *inhibited α-SMA activation, including its gene expression into mRNA and protein.

**Conclusion:**

Among the five Chinese medicine decoctions, *YCHT *exerted the most significant therapeutic effects on DMN-induced cirrhosis/fibrosis in rats.

## Background

Liver fibrosis occurs as a result of a variety of pathological factors, including viral hepatitis (especially hepatitis B and C), alcohol and drug abuse, metabolic diseases due to overload of iron or copper, autoimmunity against hepatocytes or bile duct epithelium, and congenital abnormalities [1]. Liver fibrosis, cirrhosis in particular, is associated with significant morbidity and mortality. As liver fibrosis may not manifest itself clinically until an advanced (*i.e*. cirrhotic) stage, the capability of reversing the fibrosis is highly desirable for developing therapeutic approaches [2]. At present, there is no standard treatment for liver fibrosis. Experimental studies indicated that the progression of liver fibrosis can be prevented in rodents [3], although the efficacy of most of the preventive treatments has not been established in humans. Therefore, it is imperative to develop anti-fibrotic strategies to treat liver fibrosis and cirrhosis.

Chinese medicine has been widely used for treating chronic liver hepatitis and liver cirrhosis. Chinese medicine treatment improved clinical symptoms, liver function and quality of life for patients, inhibited liver inflammation and fibrous tissue proliferation, and reversed liver fibrosis. According to Chinese medicine theories, liver cirrhosis is caused by internal damp (*Shi*) heat (*Re*), blood (*Xue*) stasis, and both *Qi *and Yin asthenia. Thus, the main Chinese medicine approach to liver cirrhosis is to eliminate heat, dispel dampness, activate blood, promote *Qi *and cultivate Yin [4-6]. In this study, five classical decoctions, namely *Yinchenhao Tang *(*YCHT*), *Xiayuxue Tang *(*XYXT*), *Huangqi Tang *(*HQT*), *Yiguan Jian *(*YGJ*) and *Xiaochaihu Tang *(*XCHT*) were used to treat liver cirrhosis induced by DMN (dimethylntrosamine) in rats.

First described in *Shanghan Lun *in 200 AD, *YCHT *consists of three medicinal herbs: *Herba Artemisiae Scopariae *(*Yinchenhao*), *Fructus Gardeniae *(*Zhizi*), and *Radix et Rhizoma Rhei *(*Dahuang*), at a weight ratio of 18:10:6 respectively. *YCHT *has been recognized by some practitioners and researchers for its beneficial effects in treating liver diseases [7]. *YCHT *was shown to ameliorate hepatitis [8] and liver fibrosis in various animal fibrotic models [9-12]. *XYXT *was first described in *Jinkui Yaolue *in the East Han Dynasty. It consists of three herbs: *Radix et Rhizoma Rhei *(*Dahuang*)*, Semen Persicae *(*Taoren*), and *Eupolyphaga Seu Steleophaga *(*Tubiechong*), at a weight ratio of 10:10:6 respectively. *XYXT *significantly inhibited liver fibrosis induced by porcine serum [13] and CCl_4 _in rats. *HQT *was first described in *Hejiju Fang*. It consists of three medicinal herbs: *Radix Astragali *(*Huangqi*), *Radix et Rhizoma Glycyrrhizae *(*Gancao*), and *Fructus Jujubae *(*Dazao*), at a weight ratio of 6:1:1. *HQT *and its components inhibited bile-duct-ligation (BDL)-induced cholestatic cirrhosis [14]. *YGJ *was first described in *Liuzhou Yihua *and consists of six herbs: *Radix Glehniae *(*Beishashen*), *Radix Ophiopogonis *(*Maimendong*), *Radix Angelicae Sinensis *(*Danggui*), *Radix Rehmanniae *(*Shengdihuang*), *Fructus Lycii *(*Gouqizi*), and *Fructus Toosendan *(*Chuanlianzi*). It was found to inhibit CCl_4_-induced cirrhosis [15]. *XCHT *consists of seven herbs: *Radix Bupleuri *(*Chaihu*), *Radix Scutellariae *(*Huangqin*),*Radix Ginseng *(*Renshen*),*Rhizoma Pinelliae *(*Banxia*),*Radix et Rhizoma Glycyrrhizae *(*Gancao*),*Rhizoma Zingiberis Recens *(*Shengjiang*),*Fructus Jujubae *(*Dazao*). It was first described in *Shanghan Lun *and has been widely used to treat chronic hepatitis in Japan since the 1970s. Its anti-fibrosis action has been reported [16].

This study aims to (1) determine which of these five decoctions is most effective to treat DMN-induced liver fibrosis; and (2) establish a molecular basis of the most effective decoction.

## Methods

### Chemicals and herbs

DMN was purchased from Sigma. Hydroxyproline (Hyp) standard was purchased from Nakateitesuku Corporation (Japan). Liver function test kit was purchased from Nanjing Jiancheng Biotech Company (China). *XCHT *(TJ-9) was purchased from Tumura Corporation (Japan). Herb constituents of *YCHT*, *XYXT*, *HQT *and *YGJ *were purchased from Shanghai Huayu Chinese Herbs Co Ltd (China). The herbs were accredited by a pharmacognosist according to standard protocols, prepared by Shuguang Hospital, and then stored at -20°C.

### Animal models of liver fibrosis

Eighty-eight male Wister rats of weight 180–200 g were kept in an air-conditioned room at 25°C with a 12 hour dark/light cycle and unlimited access to food and water. They were randomly divided into two groups: (1) control (n = 10) and (2) DMN (n = 78). DMN (10 mg/kg body weight) was administered intraperitoneally to DMN rats for three consecutive days each week for four weeks [2]; normal saline was given to control rats. At the end of week 2, three control rats and six DMN rats were sacrificed for observation. The remaining DMN rats (n = 72) were randomly divided into six groups: DMN+ water, *YCHT, XYXT, HQT, YGJ *and *XCHT *(n = 12). At the end of week 4, all rats were anesthetized with 40 mg/kg body weight sodium pentobarbital. Blood was collected from interior vena cava. The livers and spleens were immediately removed, washed with ice-cold saline, dried by filter paper, and weighed in the wet state.

### Liver function tests

Serum was collected from blood by centrifugation at 1300 × g at 4°C and stored at -80°C for liver function tests. Serum activities of alanine transaminase (ALT), aspartate transaminase (AST), alkaline phosphatase (ALP), content of albumin (Alb) and total bilirubin (TBIL) were tested. Total bile acid (TBA) was measured by a clinical laboratory of Shuguang Hospital.

### Histology examination

Liver tissues were taken from the right lobe of the liver of each rat, and fixed in 4% buffered paraformaldehyde, dehydrated in a graded alcohol series. Following xylene treatment, specimens were embedded in paraffin blocks, cut into 5 μm-thick sections and placed on glass slides. The sections were stained with hematoxylin-eosin and Sirius red respectively. Fibrosis was graded according to the method by Scheuer [17] as follows: grade 0: normal liver; grade 1: an increase of collagen without the formation of septa (small satellite expansion of the portal fields); grade 2: formation of incomplete septa not interconnecting with each other, from the portal tract to the central vein; grade 3: complete but thin septa interconnecting with each other, which divide the parenchyma into separate fragments; and grade 4: complete cirrhosis, similar to grade 3 with thicker septa. The pathological examination was performed by three pathologists who were blinded to the rats' treatment assignment. Fibrosis scores were given after the pathologists had thoroughly examined three different areas of the tissue slide for each rat.

### Hepatic hydroxyproline content

Liver tissue (100 mg) was prepared for hydroxyproline (Hyp) determination according to a modification of method by Jamall *et al*. [18]. The Hyp content of the liver as an indirect measure of tissue collagen content was expressed as microgram per gram of wet weight (μg/g).

### Expression and immunochemistry of α-SMA

Total RNA was extracted from 50 mg of liver tissue using Trizol reagent (Invitrogen, USA). Synthesis of cDNA was performed using 4 μg of total RNA per sample with random primers and reagents contained in the RevertAid First Strand cDNA synthesis kit (Fermentas, Lithuania). Two microliters (2 μl) of each sample was used for real-time PCR in a Rotor-Gene 2000 system (Australia). Relative quantitation was calculated using delta cycle threshold (CT) relative quantitation. Sequences and accession number of primers used for alpha smooth muscle actin (α-SMA) are: 5'-CGAGAGGACGTTGTTAGCATAGAG-3' (forward) and 5'-GGGCATCCACGAAACCA-3' (reverse) (GenBank accession number: BI282702; product length: 85 bp). Sequences and accession number of primers used for 18S rRNA are: 5'-GTAACCCGTTGAACCCCATT-3' (forward) and 5'-CCATCCAATCGGTAGTAGCG-3' (reverse) (GenBank accession number: X00117; product length: 151 bp).

Liver samples were prepared with Radio immunoprecipitation assay (RIPA) lysis buffer containing protease inhibitors at 4°C. After quantification, the proteins (50 μg/lane) were separated by polyacrylamide gel electrophoresis and transferred to Immobilon-P membranes which were then blocked and exposed to α-SMA (Chemicon, CBL171, 1:100, USA). The antigen was demonstrated by enhanced chemiluminescence (Pierce, USA) for one minute, followed by exposure to Kodak film (Kodak, USA).

After deparaffinization and dehydration, microwave antigen retrieval was performed for five minutes prior to peroxidase quenching with 3% H_2_O_2 _in PBS for 15 minutes. Subsequently, the sections were preblocked with 5% bovine serum albumin for 30 minutes. Slides were incubated with anti-α-SMA (Sigma, A2547, 1:400, USA) antibody overnight at 4°C and with biotinylated secondary antibodies for 45 minutes respectively. They were then developed with diaminobenzidine (DAB) for 3 minutes and finally counterstained with hematoxylin.

### Statistical analysis

All results were expressed as mean and standard deviation (SD). Measurement data were analyzed using a one-way analysis of variances (ANOVA, SPSS 11.0). Rank data were analyzed with ridit. The result of P < 0.05 was considered to be statistically significant.

## Results

### Effects on survival and liver/spleen weight ratio

During drug intervention process, two rats died in the DMN + *XCHT *group. Animal body and liver weights were monitored during the formation of liver fibrosis (Table 1). Body weights of the rats from the 2-week DMN group and 4-week DMN + water group were significantly lower than those of the control group during the same period (P < 0.01). Livers of the control rats were smooth, ruddy, and soft, while those of the 2-week DMN rats were slightly bigger, accompanied by hyperemia and edema. In the DMN + water rats, livers were much smaller and more rigid. Liver/body weight ratio of the DMN + water group was significantly lower than that of the control group (P < 0.01); spleen/body weight ratio of the DMN + water group significantly increased (P < 0.01). The liver/body weight ratio of the *YCHT *group was higher than that of the DMN + water group without significant difference. Spleen weight of the *YGJ *group was significantly lower than that of the 4-week DMN control group (P < 0.05). No significant improvements were found in the other groups.

**Table 1:** Effects of five Chinese medicine decoctions on body weight, liver weight, spleen weight, liver/body, spleen/weight in DMN induced liver fibrosis of rats

Group	Group size (n)	Body weight(g)	Liver weight (g)	Spleen weight (g)	Liver/body (%)	Spleen/body (‰)
2-week control	3	226.0 (2.64)^▲△△^	7.80 (0.26)^△△^	0.66 (0.07)^▲▲△△^	3.45 (0.12)^▲△△^	2.90 (0.28)^▲▲△△^
2-week DMN	6	175.8 (6.11)	7.02 (0.32)^▲^	1.02 (0.12)^▲▲^	3.99 (0.09)^▲▲^	5.80 (0.58)^▲^
4-week control	7	287.0 (8.98)^▲▲△△^	10.03 (0.49)^▲▲△^	0.76 (0.05)^▲▲△△^	3.49 (0.08)^▲▲△△^	2.64 (0.17)^▲▲△△^
DMN + water	12	204.2 (20.22)	5.69 (1.78)^△^	1.43 (0. 22)^△△^	2.73 (0.61)^△△^	7.27 (0.35)^△^
DMN + *YCHT*	12	197.27 (22.10)	5.91 (1.95)	1.49 (0.25)	2.93 (0.74)	7.54 (0.68)
DMN + *XYXT*	12	208.33 (15.11)	5.22 (1.42)	1.60 (0.21)	2.48 (0.54)	7.67 (0.66)
DMN + *HQT*	12	212.00 (20.20)	5.52 (1.70)	1.57 (0.20)	2.58 (0.72)	7.44 (0.87)
DMN + *YGJ*	12	193.17 (24.41)	4.50 (1.57)	1.29 (0.21)^▲^	2.28 (0.59)	6.65 (0.67)
DMN + *XCHT*	10	216.70 (17.08)	5.22 (1.16)	1.55 (0.15)	2.40 (0.42)	7.17 (0.72)

^▲^P < 0.05 ^▲▲^P < 0.01 (*vs *DMN+ water)

^△^P < 0.05 ^△△^P < 0.01 (*vs *2-week DMN)

*YCHT*: *Yinchenhao Tang*

*XYXT*: *Xiayuxue Tang*

*HQT*:*Huangqi Tang*

*YGJ*: *Yiguang Jian*

*XCHT*: *Xiaochaihu Tang*

Results are expressed as mean (SD).

### Effects on hepatic histopathological changes

**Figure 1:**
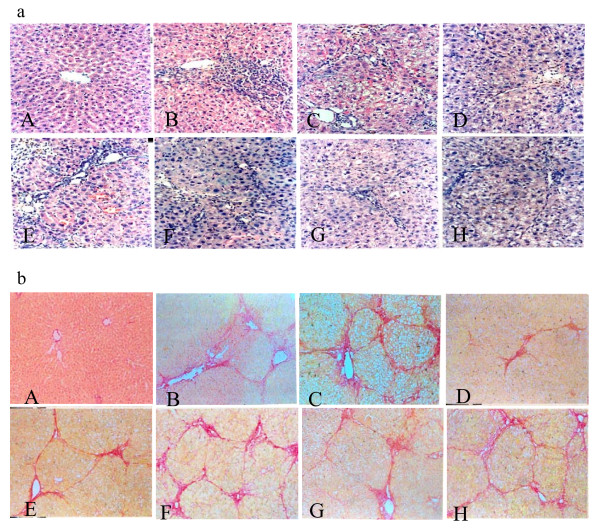
Effects of the five Chinese medicine decoctions on histological changes of liver. (a) Hematoxylin - eosin (H&E) ×200. (A) Control rats (B) 2-week DMN fibrotic rats (C) DMN + water cirrhotic rats (D) DMN + *YCHT *rats (E) DMN + *XYXT *rats (F) DMN + *HQT *rats (G) DMN + *YGJ *rats (H) DMN + *XCHT *rats. (b) Sirius red stain ×100. (A) Collagen was scarcely observed. (B) Collagen was stretched from portal to lobular area. (C) Portal and lobular bridging fibrosis, and cirrhotic nodule formation were observed. (D) Fibrosis significantly decreased. (E), (F), (G) and (H) Changes in collagen deposition were observed.

Livers in the control group showed normal lobular architecture with central vein and radiating hepatic cords (Figure 1a). After two weeks of DMN-intoxication, massive hepatocyte necrosis, intense neutrophilic infiltration, mild bile duct hyperplasia and initiation of fibrosis were found (Figure 1a). There was diffuse centrilobular congestion with marked dilatation of central veins. Extensive necrosis and hemorrhage were prominent at this early stage. In the DMN + water group, the liver sections revealed collagen deposition, marked cirrhosis, collagen fiber deposition, severe centrilobular necrosis, focal fatty changes, bile duct proliferation, bridging necrosis and apoptosis, and fibrosis surrounding the central veins (Figure 1a). In the DMN + *YCHT *group, marked reduction of the thickening of the collagen bundles was seen. In the DMN + *XYXT*/*HQT*/*YGJ*/*XCHT *groups, changes in hepatocyte necrosis and bile duct hyperplasia were shown (Figure 1a).

### Effects on liver collagen deposition

Quantitative assessment of liver fibrosis was performed with morphometry on sections processed with 0.1% Sirius red which specifically stains collagen. Collagen was scarcely observed except around the small central venous walls in normal liver (Figure 1b). In the 2-week DMN rats, collagen was seen to stretch from portal area to lobular, and incomplete septa were also observed (Figure 1b). In the DMN + water rats, livers showed marked distortion in architecture, including portal and lobular bridging fibrosis, cirrhotic nodule formation, and thickened reticulum fibers joining central areas. Normal structure disappeared, while pseudolobules formed (Figure 1b) with 75% rats with cirrhosis. Compared with the DMN + water group, fibrosis scores of livers in *YCHT *group significantly decreased (P < 0.05). Only one rat developed typical cirrhosis; the rate of cirrhosis was 8.3%. Ridit analysis showed that the DMN + *YCHT *rats were significant different from the DMN + water rats (P < 0.05). Other intervention groups were not significant different (Figure 1 and Table 2).

**Table 2:** Effects of five Chinese medicine decoctions on liver hydroxyproline (Hyp) content in DMN-induced liver fibrosis of rats

Group	Group size (n)	Hyp content μg/g mean (SD)	Fibrotic grade*					95% CI of U**
				
			0	1	2	3	4	
Control	10	179.36(9.90)^▲▲^	10	0	0	0	0	(-0.110 to 0.228)^▲▲^
2-week DMN	6	289.06 (26.95)^▲▲^	0	0	4	2	0	(0.051 to 0.486)^▲▲^
DMN + water	12	641.71 (54.22)^△△^	0	0	0	3	9	(0.586 to 0.894)^△△^
DMN + *YCHT*	12	434.16 (25.39)^▲^	0	0	5	6	1	(0.226 to 0.548)^▲^
DMN + *XYXT*	12	586.91 (37.53)	0	0	0	5	7	(0.522 to 0.829)
DMN + *HQT*	12	559.04 (47.06)	0	0	0	8	4	(0.580 to 0.734)
DMN + *YGJ*	12	647.17 (64.73)	0	0	1	6	5	(0.435 to 0.743)
DMN + *XCHT*	10	636.35 (48.53)	0	0	1	7	2	(0.333 to 0.671)

*: grade 0: normal; grade 1: very slight; grade 2: slight; grade 3: moderate; grade 4: severe. Data are expressed as numbers of animals with each fibrotic grade.

**: 95% confidence interval of the U values obtained from ridit analysis

^▲^P < 0.05 ^▲▲^P < 0.01 (*vs *DMN+ water)

^△^P < 0.05 ^△△^P < 0.01 (*vs *2-week DMN)

*YCHT*: *Yinchenhao Tang*

*XYXT*: *Xiayuxue Tang*

*HQT*:*Huangqi Tang*

*YGJ*: *Yiguang Jian*

*XCHT*: *Xiaochaihu Tang*

Results are expressed as mean (SD).

### Effects on hepatic hydroxyproline content

Changes in Hyp content in the liver are considered an index for collagen metabolism and provide valuable information about the biochemical and pathological states of liver fibrosis. Liver collagen content, expressed as microgram (μg) of Hyp/gram (g) of liver tissue, is shown in Table 2. The Hyp content in the DMN rats was approximately 161% of that of the control group (P < 0.01) at the end of week 2, suggesting abundant accumulation of collagen in the livers of DMN rats. The highest increase, which was about 3.6-fold compared with the control (P < 0.01), was observed at the end of week 4 in the DMN rats + water group. It was consistent with the observation of marked cirrhosis and accumulation of collagen bundles in the liver by histopathological examination. There was a significant decrease (P < 0.05) in liver Hyp content (about 41% in the DMN + water rats) in the *YCHT *rats, suggesting that *YCHT *ameliorated hepatic collagen deposition in DMN-induced liver injury. Hyp content in the DMN + *HQT*/*XYXT *rat livers was slightly reduced without significant difference. Hyp content in DMN + *XCHT*/*YGJ *rats was not changed.

### Effects on liver function

Liver function parameters deteriorated over time in rats subjected to DMN (Table 1). In DMN rats, ALT, AST, ALP, TBIL and TBA levels were significantly higher after two and four weeks compared with the control rats (P < 0.01), indicating hepatic injury. ALT, AST, ALP, TBIL and TBA levels also increased markedly in the cirrhotic DMN + water rats. Oral administrations of *YCHT *reduced the DMN-induced increase in ALT, AST, ALP activities as well as TBIL and TBA content (P < 0.01). These decreased biochemical parameters in the DMN + water rats indicated that *YCHT *ameliorated hepatic injury in the DMN rats. Serum Alb content in the 2-week and 4-week DMN rats was significantly lower than that in the control rats. *YCHT *could elevate Alb content, but there was no significant difference compared with the DMN + water group (Table 3).

**Table 3:** Effects of five Chinese medicine decoctions on serum activities of AST, ALT and ALP and serum content of TBA and TBIL in DMN-induced liver fibrosis of rats

Group	ALT(U/L)	AST(U/L)	ALP (K/100 ml)	TBA(μmol/L)	TBIL (g/L)	Alb (g/L)
Control	20.13 (3.10)^▲▲△△^	28.03 (4.33)^▲▲△△^	33.73 (4.95)^▲▲△△^	19.30 (6.03)^▲▲△△^	11.5 (0.93)^▲▲△△^	40.89 (2.23)^▲▲△△^
2 week DMN	40.62 (4.96)^▲▲^	42.08 (2.35)^▲▲^	56.85 (9.80)^▲▲^	23.5 (8.06)^▲▲^	20.0 (6.52)^▲▲^	35.06 (2.27)^▲▲^
DMN + water	70.30 (6.38)^△△^	81.08 (3.18)^△△^	97.67 (3.58)^△△^	56.8 (9.45)^△△^	24.1 (7.71)^△△^	26.38 (4.11)^△△^
DMN + *YCHT*	39.34 (5.87)^▲▲^	45.39 (5.16)^▲▲^	77.20 (5.28)^▲▲^	38.1 (10.08)^▲▲^	16.7 (4.68)^▲^	27.06 (4.16)
DMN + *XYXT*	60.26 (6.70)^▲▲^	70.89 (5.72)	83.55 (13.26)^▲▲^	38.0 (12.90)^▲▲^	16.9 (6.68)^▲^	26.74 (4.71)
DMN + *HQT*	58. 23 (0.80)^▲▲^	66.12 (5.58)^▲▲^	94.19 (6.35)	40.8 (10.47)^▲▲^	19.0 (2.36)	28.89 (2.20)^▲^
DMN + *YGJ*	66.77 (5.29)	75.09 (2.25)	79.37 (3.39)^▲▲^	47.0 (21.02)	20.0 (15.19)	26.13 (3.67)
DMN + *XCHT*	68.41 (5.82)	77.42 (5.59)	93.38 (1.98)	55.3 (14.34)	23.0 (13.52)	25.88 (2.95)

^▲^P < 0.05 ^▲▲^P < 0.01 (*vs *DMN+ water)

^△^P < 0.05 ^△△^P < 0.01 (*vs *2-week DMN)

*YCHT*: *Yinchenhao Tang*

*XYXT*: *Xiayuxue Tang*

*HQT*:*Huangqi Tang*

*YGJ*: *Yiguang Jian*

*XCHT*: *Xiaochaihu Tang*

Results are expressed as mean (SD).

### Effects of YCHT on the gene expression of α-SMA into mRNA and protein

In liver function test, histopatholgical observation and hepatic Hyp content detection, *YCHT *showed a comprehensive inhibitory effect on DMN-induced liver fibrosis. We went on to detect the anti-fibrotic function of *YCHT *using molecular biology techniques.

**Figure 2:**
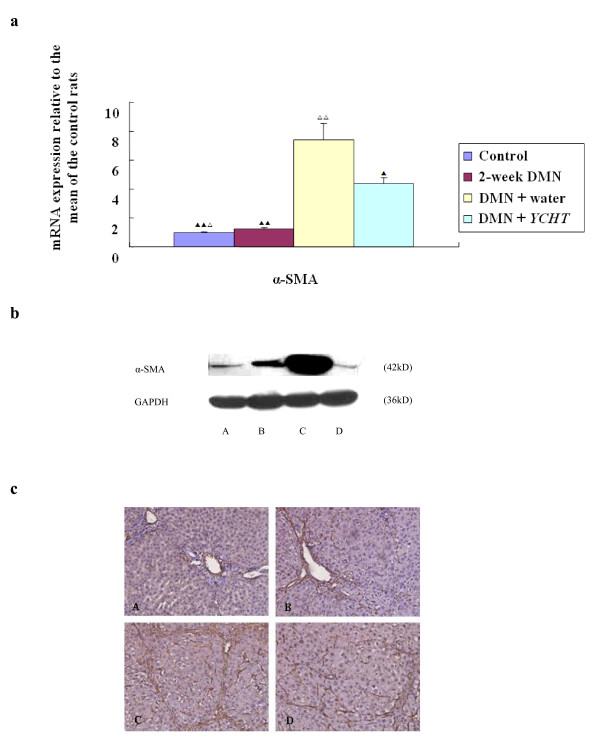
(a) Real-time quantitative PCR. In the 2-week DMN group, α-SMA mRNA expression level increased (*vs *control rats, P < 0.01). In the DMN + water group, α-SMA mRNA expression level also increased (*vs *control rats, P < 0.01). In the DMN + *YCHT *group, α-SMA mRNA expression level decreased (*vs *DMN + water rats, P < 0.05). ^▲^P < 0.05 ^▲▲ ^P < 0.01 (*vs *DMN+ water) ^△ ^P < 0.05 ^△△ ^P < 0.01 (*vs *2-week DMN). (b) Western blot. Western blot analysis of total protein extracts was performed with antibodies recognizing α-SMA and Glyceraldehyde 3-phosphate dehydrogenase (GAPDH). *YCHT *reduced α-SMA protein expression significantly (*vs *DMN + water). (c) Immunohistochemical analysis ×200. (A) In the control group, α-SMA-positive HSCs were weakly stained and only detected in vascular structures. (B) and (C) With DMN-intoxication, number of α-SMA-positive HSCs increased gradually and peaked at week 4. (D) In the DMN + *YCHT *group, number of α-SMA-positive HSCs was significantly reduced.

Levels of α-SMA mRNA increased dramatically in the DMN rats at week 2 (P < 0.01) (Figure 2a). They also increased remarkably in the DMN + water group (P < 0.01). In the DMN + *YCHT *group, α-SMA mRNA expression decreased remarkably (P < 0.05) compared with the DMN + water group. The result was also confirmed by immunoblotting (Figure 2b).

In the control rats, α-SMA-positive hepatic stellate cells (HSCs) were weakly stained and were only detected in vascular structures (Figure 2c). Along with DMN-intoxication, the number of α-SMA-positive HSCs increased gradually and reached peak at week 4 in cirrhotic livers (Figure 2c). In the DMN + *YCHT *group, number of α-SMA-positive HSCs was significantly reduced (Figure 2c).

## Discussion

DMN-induced liver fibrosis in rats is a reproducible model for studying the pathogenesis of liver fibrosis and cirrhosis [19,20]. DMN-intoxication for four weeks produced centrilobular necrosis and fully-developed cirrhosis in rats. The approximately four-fold increase of total liver collagen observed in the present study is consistent with the previous investigations on DMN-induced liver fibrosis in rats [21-24]. Two weeks of DMN-intoxication produced diffuse centrilobular congestion with marked dilatation of central veins. Extensive necrosis and hemorrhage were prominent at this early stage; all rats developed liver fibrosis of grades 2–3. Liver Hyp content significantly increased 1.61-fold as compared with the normal rats. Notable abnormality in liver function was also observed. In the DMN + water group, liver damage significantly increased; liver weight notably reduced and splenomegaly was observed. Liver structure became disordered seriously with rapid progress of fibrosis [25-27]. Hyp content in liver tissue of the DMN + water group, which increased 3.68-fold, was much higher than that of the control group. Seventy-five per cent (75%) of the DMN + water group have developed cirrhosis. This study related to the common symptoms of liver cirrhosis as well as its pathogenesis in traditional Chinese medicine. Five decoctions were chosen to prevent the rapid formation phase of liver cirrhosis from fibrosis. This study revealed different efficacies of the five decoctions. The results showed that *YCHT *had some specific therapeutic effects on pathological changes in the liver. Liver weight reduction was stopped and liver function was improved; serum activities of ALT, AST and ALP were restrained at basic level for the 2-week DMN rats; serum TBIL content was lowered for 2-week DMN rats. Liver cell degeneration, necrosis, inflammation and infiltration were significantly reduced. Liver fibrous tissue hyperplasia and liver structural alterations were markedly inhibited. Only one of the 12 rats had liver cirrhosis, cirrhosis formation rate being 8.3%. Hyp content was 44.9%, lower than that of the DMN + water group. The other four decoctions did not show these significant effects. Hepatocyte necrosis, sinusoidal endothelial injury, inflammation and bleeding induced by DMN were considered to be the major factors for the development of liver fibrosis. Sustained inflammation caused extracellular matrix deposition and nodular regenerative transition [16,28].

Activation of HSCs is a key factor in developing liver fibrosis. Inhibition of the accumulation of activated HSCs by modulating either their activation and/or proliferation or promoting their apoptosis is thus an important therapeutic strategy. *YCHT *reduced α-SMA (a marker of activated HSCs [29]) expression at mRNA and protein synthesis levels. Our results indicated that the molecular mechanism of anti-fibrotic effects by *YCHT *might be due to the suppression of HSCs' activation.

In another study by our team, we used the same decoctions for treating liver cirrhosis induced by DMN in rats at the cirrhosis formation stage. The rats were injected with DMN for four weeks and were treated with the herbal medicines for two weeks. Among the five decoctions, both *YCHT *and *HQT *exerted significant therapeutic effects and *YCHT *was overall more effective than *HQT *[29]. The present study demonstrated the effectiveness of *YCHT *in DMN-induced rat liver fibrosis/cirrhosis and pathological changes in different stages of the disease. In this study, rats were exposed to DMN and received herbal medicine intervention after the formation of liver fibrosis after two weeks of DMN-intoxication. In general no significant effect of intervention by *HQT *was found; although it was previously found to be effective in DMN-induced liver cirrhosis. It is probable that the rats which were exposed to DMN sustained as long as intervention therapy was given. However, when liver cirrhosis was formed and pathogenic factors were removed for two weeks, inflammatory injury could be relaxed gradually. Hepatic parenchymal cell injury appears gradually in this period. Thus, pathological changes in various stages of liver fibrosis and cirrhosis may affect the efficacy of the decoctions.

## Conclusion

Among the five Chinese medicine decoctions, *YCHT *demonstrated the most significant anti-fibrotic effects on DMN-induced liver cirrhosis/fibrosis in rats, which may be a result of the decoction's inhibitory effects on HSCs' activation.

## Abbreviations

α-SMA: α-smooth muscle actin; Alb: albumin; ALP: alkaline phosphatase; ALT: alanine transaminase; AST: aspartate transaminase; CT: cycle threshold; DAB: diaminobenzidine; DMN: dimethylnitrosamine; HSCs: hepatic stellate cells; Hyp: hydroxyproline; RIPA: radioimmunoprecipitation assay; TBA: total bilirubin acid

## Competing interests

The author(s) declare that they have no competing interests.

## Authors' contributions

CL and PL conceived the study design. CL, LW, GW and GC performed the data analysis. CL and MS drafted the manuscript. CHL and PL revised the manuscript. All authors confirm that the content of this paper has not been published elsewhere and does not overlap or duplicate their published work. All authors read and approved the final manuscript.
